# *Chlamydia trachomatis* impairs T cell priming by inducing dendritic cell death

**DOI:** 10.1128/iai.00402-24

**Published:** 2025-01-08

**Authors:** Haitong Mao, Eric K. Dumas, Michael N. Starnbach

**Affiliations:** ^1^Department of Microbiology, Harvard Medical School 1811 , Boston, Massachusetts, USA; University of Pennsylvania Perelman School of Medicine, Philadelphia, Pennsylvania, USA

**Keywords:** *Chlamydia*, T cells, dendritic cells

## Abstract

The lack of effective adaptive immunity against *Chlamydia trachomatis* leads to chronic or repeated infection and serious disease sequelae. Dendritic cells (DCs) are professional antigen-presenting cells that are crucial for the activation of T cells during *C. trachomatis* infection. cDC1s and cDC2s are the two main DC subsets responsible for T cell priming, but little is known about how *C. trachomatis* affects their ability to prime T cells. Using a mouse model of infection, we found that *C. trachomatis* uptake reduced the viability of cDC1s and cDC2s both *in vitro* and *in vivo*, with cDC1s experiencing more death. DC death was mainly due to apoptosis and is alleviated in *Casp3/7* or *Bak1/Bax* knockout DCs. In addition, we observed that *C. trachomatis*-specific CD8+ T cells were preferentially activated by cDC1s. Reduction in DC viability by *C. trachomatis* impaired the ability of infected DCs to activate T cells upon co-culture, although in the case of CD8+ T cell priming, controlling for viability was insufficient to fully rescue the defect. RNA sequencing of DCs from infected mice showed upregulation of cell death pathways, supporting our observations of DC death caused by *C. trachomatis*. Finally, we validated our findings with human DCs *in vitro*, observing *C. trachomatis*-induced cell death. These results indicate that *C. trachomatis* may evade the adaptive immune system by directly inducing cell death in DCs.

## INTRODUCTION

*Chlamydia trachomatis* is an obligate intracellular bacterium that infects epithelial cells. It is the most common bacterial sexually transmitted disease in the United States, with an estimated 2.86 million infections occurring annually (1). While curable with antibiotics, infections are often asymptomatic and thus left untreated (2). This can lead to serious sequelae in women, such as pelvic inflammatory disease, infertility, and ectopic pregnancy (3). Despite the high public health burden of *C. trachomatis*, a vaccine to prevent this disease is not available. A better understanding of the immune response to *C. trachomatis* is, therefore, crucial to the development of an effective vaccine (4).

In humans, the immune system is frequently incapable of clearing *C. trachomatis* infection or establishing long-term immunological memory, resulting in chronic or repeated infections (5). In contrast, mice are able to clear infection in a CD4+ T cell-dependent manner (6, 7), but an efficient CD8+ T cell response is not generated (8, 9). This is especially intriguing since *C. trachomatis* is an intracellular pathogen, and CD8+ T cells typically play an important role in protecting against such organisms by killing the cells they infect. *C. trachomatis*-specific CD8+ T cells are primed during infection (10, 11), although at much lower numbers compared to model intracellular pathogens like vaccinia virus or *Listeria monocytogenes* (8). However, when cultured and adoptively transferred, *Chlamydia-*specific CD8+ T cells do confer partial protection against infection (10). These data suggest that antigen-specific CD8+ T cells are able to protect against *C. trachomatis*, but they are not efficiently generated during natural infection.

Dendritic cells (DCs) are the most important antigen-presenting cells (APCs) in stimulating T cell responses during infection (12, 13). In response to infection, DCs transit from an immature state to a mature state, where they upregulate co-stimulatory molecules and produce inflammatory cytokines to stimulate effector T cell responses (14). Meanwhile, in tolerogenic conditions, DCs can prevent effector T cell formation through the upregulation of co-inhibitory molecules (15, 16). There are two main subsets of DCs responsible for antigen presentation: cDC1s, which can be identified in mice as being CD103+ in the periphery and CD8+ in secondary lymphoid organs, and cDC2s, which are CD11b+ (17). These DC subsets have distinct transcriptional profiles and have varying antigen presentation capacity. In both cases, antigens are presented to T cells as peptides bound to major histocompatibility complex (MHC) class I or class II molecules. Traditional CD8+ T cells recognize peptide antigens presented on MHC I. In most cells, only cytosolic proteins are processed and presented on MHC I (18). Exogenous antigens taken up by APCs instead enter the phagolysosomal pathway where they are degraded and loaded onto MHC II for presentation to CD4+ T cells (19). Cross-presentation, where exogenous antigens are taken up and processed within the MHC I pathway, is thus required to generate CD8+ T cell responses to intracellular pathogens that do not productively infect DCs or other professional APCs (18). cDC1s are traditionally thought to be the major DC subset responsible for cross-presentation, as mice lacking cDC1s have been shown to be deficient in priming CD8+ T cells in various infection (20, 21) and tumor models (21).

Since *C. trachomatis* is an intracellular pathogen, if cDC1s are more effective than cDC2s in activating *C. trachomatis-*specific CD8+ T cells, a differential response between the two DC subsets upon infection—either in terms of survival or expression of co-inhibitory and co-stimulatory molecules—may contribute to the lack of an effective CD8+ T cell response generated against *C. trachomatis* compared to CD4+ T cells. However, studies investigating whether productive *C. trachomatis* infection occurs in DCs (22–24) or how DCs respond to *C. trachomatis* (25–29) have shown mixed results. This is further confounded by the fact that monocyte- or bone marrow-derived DCs (BMDCs) generated using GMCSF, which have been shown to be distinct from DCs *in vivo* and do not form subsets (30, 31), were used in most studies. It is, therefore, unknown how the two DC subsets respond to *C. trachomatis*, and whether cross-presentation by cDC1s is required for the activation of *C. trachomatis*-specific CD8+ T cells. In this study, we investigate how cDC1s and cDC2s respond to *C. trachomatis* and prime T cells *in vivo* and *in vitro*. We show that cDC1s are much more efficient at stimulating *C. trachomatis*-specific CD8+ T cells. *C. trachomatis* exposure decreases the T cell-priming ability of DCs via the induction of apoptosis, with the activation of CD8+ T cells more impaired than CD4+ T cells.

## RESULTS

### *C. trachomatis* infection induces dendritic cell death

**Fig 1:**
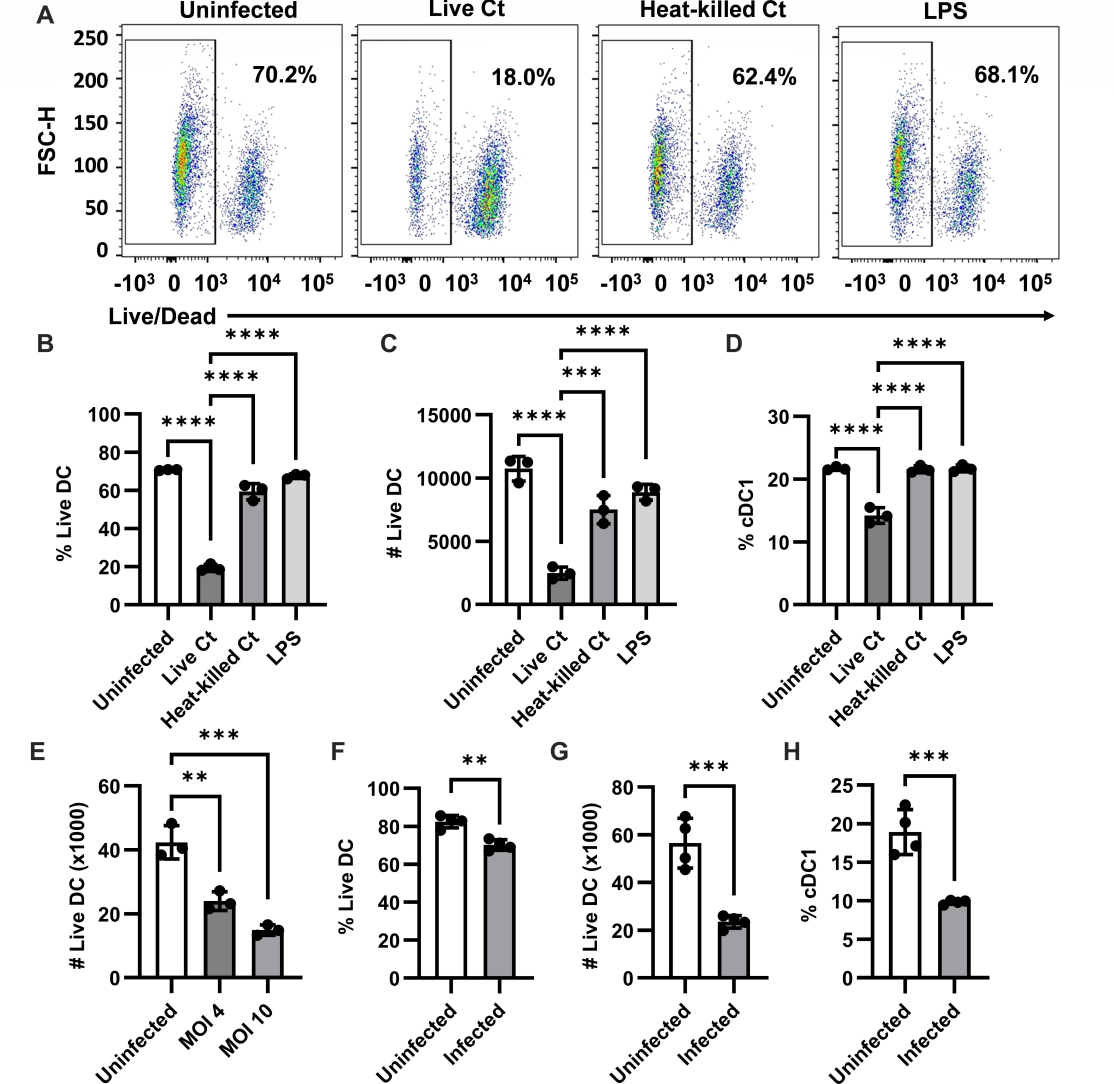
Live *C. trachomatis* induces dendritic cell death. (A–D) Splenocytes were isolated from C57BL/6 mice and infected with live or heat-killed *C. trachomatis* at an MOI of 2, or LPS control (100 ng/mL). Eighteen hours post-infection, (A and B) the percentage of viable DCs (gated on F4/80− CD11c+ MHC I-Ab+ cells), (C) number of viable DCs, and (D)percentage of viable DCs that were cDC1s (CD8+ CD11b−) were measured by flow cytometry. (E) CD11c+ dendritic cells were isolated from the spleens of C57BL/6 mice by MACS and infected with *C. trachomatis*. The viability of DCs was determined by flow cytometry. (F–H) C57BL/6 mice were infected intravenously with 10^7^ inclusion-forming units of *C. trachomatis*. Two days post-infection, (F) the percentage viability of DCs, (G) the number of viable DCs, and (H) the percentage of viable DCs that were cDC1s were measured by flow cytometry. (A–E) *n* = 3 or (F–H) *n* = 4. Data are representative of at least three independent experiments for all panels. **P < 0.01, ***P < 0.001, and ****P < 0.0001, analyzed with (B–E) one-way ANOVA and Dunnett’s multiple comparisons test or (F–H) unpaired *t* test. Data are represented as mean ± SD.

To characterize the effects of *C. trachomatis* infection on DCs, we first harvested splenocytes from wild-type (WT) C57BL/6 (B6) mice as a source of DCs. Splenocytes were infected with live or heat-killed *C. trachomatis* for 18 hours—the time point at which DCs from infected mice have picked up antigens and migrate to lymph nodes to present to T cells (25)—before being analyzed using flow cytometry (Fig. S1A). We observed a striking decrease in the viability of DCs infected with live *C. trachomatis* (Fig. 1A through C). This effect was attenuated with heat-killed *C. trachomatis* and was not a result of DC activation since LPS-treated control did not show the same cell death. In addition, this effect is specific to DCs since other cells in culture did not show the same decrease in viability when incubated with live *C. trachomatis* (Fig. S1B). In the samples infected with live *C. trachomatis*, a smaller percentage of viable DCs were cDC1s (and not cDC2s) compared to the rest of the groups (Fig. 1D), indicating that a higher percentage of cDC1s were killed compared to cDC2s. Donado et al. (32) previously found that invariant natural killer T cells can induce cell death in *C. trachomatis*-infected BMDCs. To determine if *C. trachomatis*-induced splenic DC death is dependent on other cell types, we isolated CD11c+ DCs from splenocytes before culturing them with *C. trachomatis*. Upon infection, isolated CD11c+ DCs also showed a decrease in viability (Fig. 1E).

We then sought to determine if the DC death observed *in vitro* could also be observed *in vivo*. To do this, we infected WT mice intravenously with *C. trachomatis* and analyzed the viability of splenic DCs after infection. As we observed *in vitro*, there was a decreased percentage (Fig. 1F) and number (Fig. 1G) of viable DCs in the spleens of infected mice compared to uninfected mice. The decrease in the percentage of viable DCs was smaller than what was observed *in vitro*, which may be due to clearance of dying cells *in vivo*. Also similar to what we saw *in vitro*, a smaller percentage of viable splenic DCs were cDC1s in infected mice compared to uninfected controls (Fig. 1H). These data indicate that *C. trachomatis* induces DC death both *in vivo* and *in vitro*, and cDC1s are more affected than cDC2s.

### Dendritic cell death is directly induced by *C. trachomatis*

**Fig 2:**
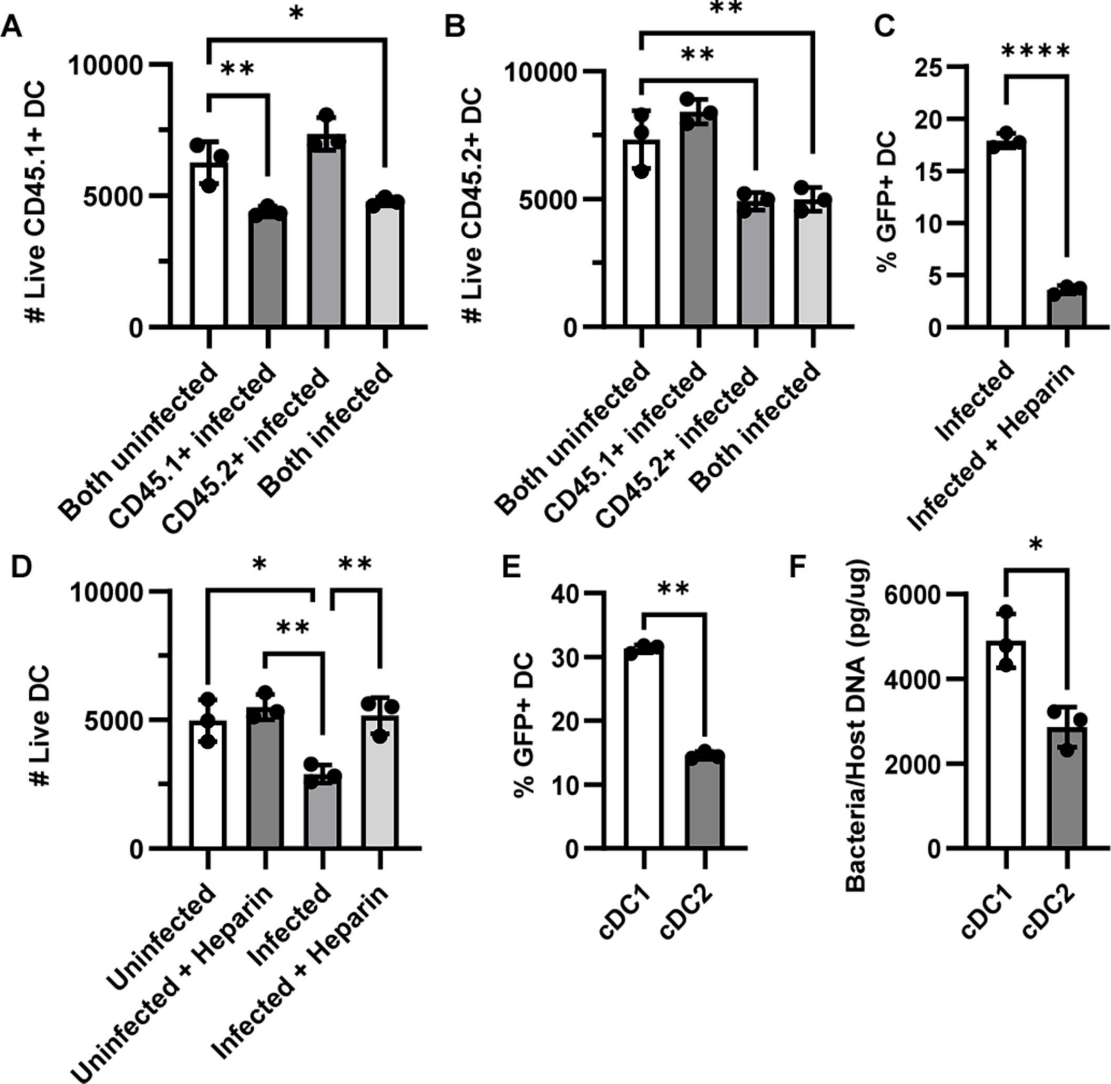
Dendritic cell death is directly induced by *C. trachomatis*. (**A and B**) CD45.1+ splenocytes were isolated from Pep Boy mice, while CD45.2+ splenocytes were isolated from C57BL/6 mice. Cells were infected with *C. trachomatis* for 2 hours, washed, and co-cultured with either infected or uninfected counterparts for 18 hours. The number of live DCs was measured by flow cytometry. (**C and D**) GFP-expressing *C. trachomatis* were pre-incubated with heparin for 1 hour before co-culturing with C57BL/6 splenocytes. (**C**) The percentage of DCs that have taken up GFP-*C. trachomatis*. (**D**) The number of viable DCs. (**E**) Splenocytes were infected with GFP-expressing *C. trachomatis*. The percentage of GFP+ cDC1s and cDC2s was measured via flow cytometry 18 hours later. (**F**) Splenic DCs were isolated with CD11c microbeads and incubated with C. *trachomatis*. Cells were then sorted into subsets by FACS. DNA was extracted, and bacteria uptake was quantified using qPCR measuring *C. trachomatis* 16s and mouse GAPDH DNA. *n* = 3, and data are representative of three independent experiments for all panels. **A, B,** and **D** were analyzed with one-way ANOVA, and **A and B** with Dunnett’s or **D **with Tukey’s multiple comparisons test; **C** with unpaired *t* test, and **E and F** with paired *t* test. *P < 0.05, **P < 0.01, and ****P < 0.0001. Data are represented as mean ± SD.

To determine whether DC death was directly induced by *C. trachomatis* or neighboring infected cells, we utilized B6.SJL-Ptprc^a^ Pepc^b^/BoyJ (Pep Boy) mice, a B6 congenic strain that expresses CD45.1 instead of CD45.2. Splenocytes were isolated from Pep Boy and B6 mice and incubated with *C. trachomatis* for 2 hours before splenocytes were centrifuged to remove bacteria that were not taken up by cells. Infected cells were co-cultured in fresh media with either infected or uninfected cells with the opposing CD45 marker for another 18 hours, and DC viability was measured. We found that only DCs directly exposed to *C. trachomatis* had a decrease in viability, and uninfected DCs cultured with infected cells were just as viable as uninfected controls (Fig. 2A and B). Since direct exposure of DCs to *C. trachomatis* was required to induce DC death, we then sought to investigate if cell death was due to *C. trachomatis* uptake. Previous studies have found that the uptake of *C. trachomatis* by cells is blocked by the pre-incubation of *C. trachomatis* with heparin (33, 34). We thus infected splenocytes with GFP-expressing *C. trachomatis* that were pre-incubated with heparin and saw that there was indeed a decrease in the percentage of DCs that had taken up *C. trachomatis* (GFP+) (Fig. 2C). Correspondingly, this pre-treatment of *C. trachomatis* with heparin also prevented DC death (Fig. 2D).

Given that cDC1s were more affected than cDC2s by *C. trachomatis*-induced cell death, we tested whether this was because cDC1s had greater uptake of *C. trachomatis* than cDC2s. We infected splenocytes with GFP-expressing *C. trachomatis* and found that a greater percentage of cDC1s had taken up bacteria compared to cDC2s (Fig. 2E). This was confirmed by qPCR, where we sorted infected splenic DCs into cDC1s and cDC2s via FACS, extracted DNA from the cells, and measured the relative levels of *C. trachomatis* 16S DNA (Fig. 2F). Taken together, our data indicate that DC death is induced by *C. trachomatis* uptake, and cDC1s have a greater uptake of *C. trachomatis* and correspondingly more death.

### Apoptosis is a major mode of *C. trachomatis*-induced dendritic cell death

**Fig 3:**
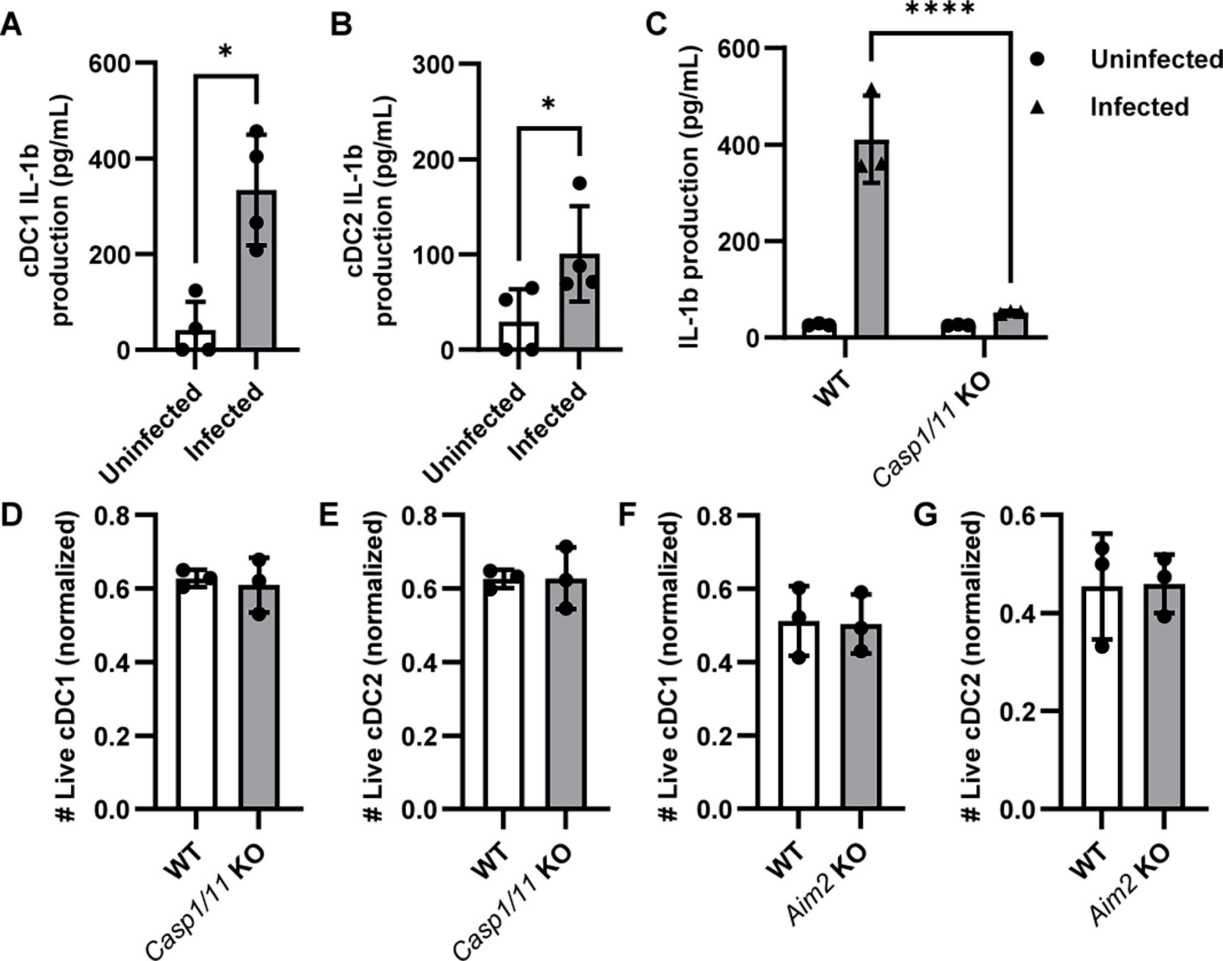
Preventing pyroptosis does not rescue *C. trachomatis*-induced cell death. (**A and B**) CD11c+ dendritic cells were isolated from the spleens of C57BL/6 mice, sorted into subsets, and infected with *C. trachomatis*. Eighteen hours post-infection, culture media were harvested, and IL-1β production by (**A**) cDC1s and (**B**) cDC2s were measured with ELISA. *n* = 4. Data were pooled from three independent experiments and analyzed with paired *t* test. (**C–E**) CD11c+ splenic dendritic cells were isolated from WT and *Casp1/11* KO mice and infected with *C. trachomatis*. (**C**) IL-1β production was measured with ELISA, and viability of (**D**) cDC1s and (**E**) cDC2s (normalized to uninfected controls from the same mouse to account for the differences in the number of live DCs between mice at steady state) determined by flow cytometry. (**F and G**) Splenic cells were isolated from WT and *Aim2* KO mice and co-cultured with *C. trachomatis*. The number of (**F**) cDC1s and (**G**) cDC2s that remained viable was determined by flow cytometry and normalized to uninfected samples from the same mouse. (**C–G**) *n* = 3, and data are representative of at least two independent experiments. **C** was analyzed with two-way ANOVA and Sidak’s multiple comparisons test, and (**D–G**) with unpaired *t* test. *P < 0.05, **P < 0.01, and ****P < 0.0001. Data are represented as mean ± SD.

We next sought to determine possible mechanisms by which *C. trachomatis* induces DC death. DCs can undergo pyroptosis, an inflammatory mode of cell death, upon detection of an intracellular pathogen. To determine whether pyroptosis was responsible for the *C. trachomatis*-induced DC death we observed, we infected cDC1s and cDC2s with *C. trachomatis* and measured IL-1β production—a hallmark of pyroptosis—in the supernatant by ELISA. Both infected cDC1s and cDC2s showed an increased production of IL-1β compared to uninfected controls (Fig. 3A and B). Caspases-1 and 11 are important effectors of pyroptosis (35). To further study the role of pyroptosis, we harvested and infected splenocytes from *Casp1/11* double knockout mice. Interestingly, *Casp1/11* double knockout prevented IL-1β production upon infection (Fig. 3C) but had no effect on the viability (Fig. 3D and E) of infected cells. *C. trachomatis* infection at high MOIs can be detected by AIM2 in macrophages (36), which activates caspase-1 in cells upon bacterial infection but is also known to drive caspase-3-dependent apoptosis—a less inflammatory mode of cell death—in caspase-1-deficient cells (37). Hence, we infected splenocytes from *Aim2* knockout mice to determine if AIM2 plays a role in *C. trachomatis*-induced DC death. No difference was observed between the viability of infected WT and *Aim2* knockout DCs (Fig. 3F and G). These data indicate that while IL-1β is produced upon infection through the activation of caspases-1 and 11, pyroptosis may not be a major mode of cell death since blocking pyroptosis does not rescue DC viability.

**Fig 4:**
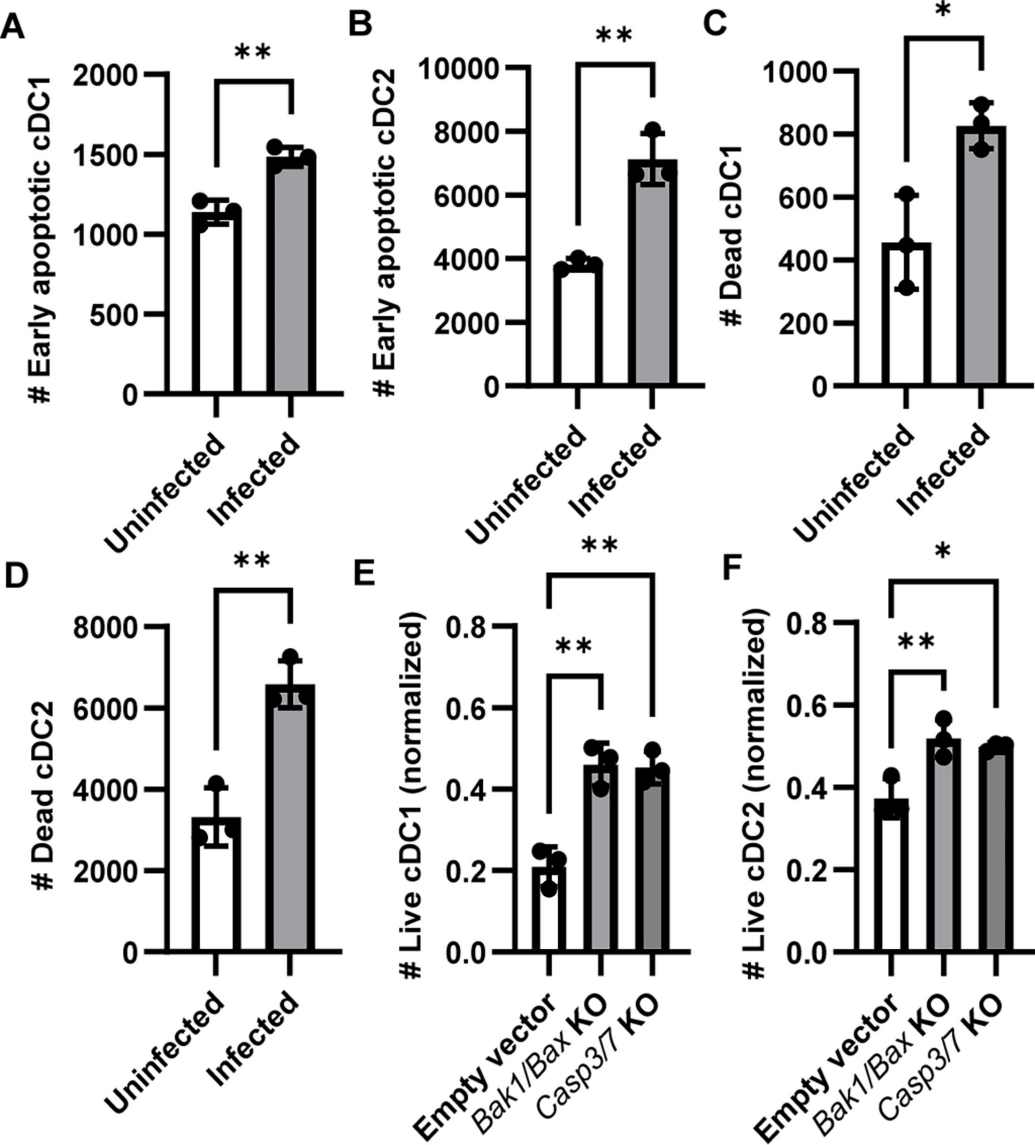
Apoptosis is a major mode *C. trachomatis*-induced dendritic cell death. (**A–D**) Splenic cells were isolated from C57BL/6 mice and co-cultured with *C. trachomatis*. After 18 hours, samples were stained with FITC-Annexin V and PI. (**A**) The number of early apoptotic cDC1s (Annexin V+ PI−) and (**B**) cDC2s. (**C**) The number of dead cDC1s (Annexin V+ PI+ ) and (**D**) cDC2s. *n* = 3, and data are representative of three independent experiments and analyzed with unpaired *t* test. (**E and F**) Bone marrow chimeras with hematopoietic cells that have *Bak1/Bax* or *Casp3/7* knocked out via CRISPR/Cas9 were created using CHIME. Eight weeks later, splenocytes were harvested and infected with *C. trachomatis* at an MOI of 1. The number of (E) cDC1s and (F) cDC2s that remained viable was determined by flow cytometry and normalized to uninfected samples from the same mouse. *n* = 3, and data are representative of two independent experiments, analyzed with one-way ANOVA and Dunnett’s multiple comparison test. *P < 0.05 and **P < 0.01. Data are represented as mean ± SD.

We then investigated if *C. trachomatis*-induced DC death was due to apoptosis. Splenocytes were infected with *C. trachomatis* and stained with Annexin V and propidium iodide (PI) before analysis with flow cytometry. After infection, we saw an increased number of early apoptotic (Annexin V+ PI−) cDC1s and cDC2s (Fig. 4A and B; Fig. S1C), suggesting that *C. trachomatis* induces apoptosis in DCs. There was also an increased number of Annexin V+ PI+ DCs that could either be late apoptotic or dead by other mechanisms (Fig. 4C and D). We next determined if blocking apoptosis could rescue *C. trachomatis*-induced DC death. Apoptosis can be prevented by deleting pro-apoptotic genes *Bak1* and *Bax* (38) or the downstream executioner caspases *Casp3* and *Casp7* (39). However, it is difficult to work with mouse models of apoptosis resistance since the majority of *Bak1/Bax* and *Casp3/7* double knockouts die perinatally (39, 40). To obtain apoptosis-resistant splenic DCs, we made use of chimeric immune editing (CHIME) (41, 42) to generate bone marrow chimeric mice with apoptosis-resistant hematopoietic cells. *Bak1/Bax* or *Casp3/7* in bone marrow stem cells were knocked out by CRISPR/Cas9 and transferred into lethally irradiated WT mice. Eight weeks after transfer, splenocytes from bone marrow chimeras were harvested and infected with *C. trachomatis*. We found that DCs lacking *Bak1/Bax* or *Casp3/7* showed increased viability upon infection compared to the WT control (Fig. 4E and F), although infection still caused a decrease in viability compared to uninfected samples. Overall, these results suggest that *Bak1/Bax* and *Casp3/7*-dependent apoptosis is the main pathway of *C. trachomatis*-induced DC death.

### RNA sequencing reveals increased cell death in dendritic cells upon infection

**Fig 5:**
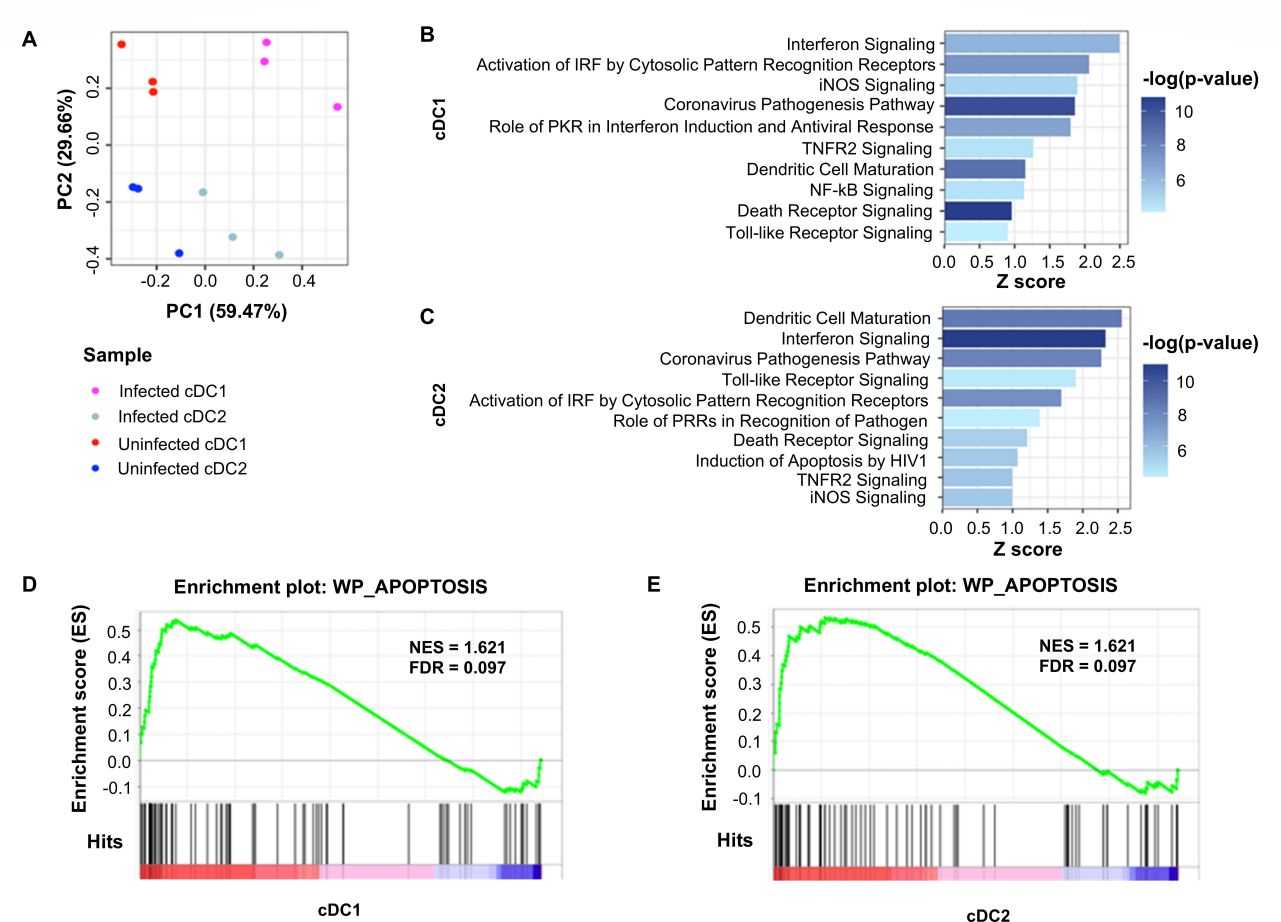
RNA sequencing reveals increased cell death in dendritic cells upon infection. C57BL/6 mice were infected with 10^7^ inclusion-forming units of *C. trachomatis* intravenously. Eighteen hours post-infection, splenic DCs were sorted into subsets, and gene expression was analyzed with RNA sequencing. (**A**) Principal component analysis of samples. (**B and C**) Top 10 pathways upregulated in infected (**B**) cDC1s and (**C**) cDC2s identified by IPA. *n* = 3, and data were analyzed using *t* test with Benjamini-Hochberg FDR correction. (**D and E**) Gene set enrichment analysis of (**D**) cDC1s and (**E**) cDC2s. Red denotes positive correlation with infected cells, while blue denotes negative correlation.

To further investigate the mechanisms of DC death upon infection, we conducted RNA sequencing to identify differences between infected and uninfected DCs *in vivo*. C57BL/6 mice were infected intravenously with *C. trachomatis*, and 18 hours post-infection, the spleens of infected mice and uninfected controls were harvested and processed for flow cytometry. Equivalent numbers of cDC1s and cDC2s were double sorted and subjected to RNA sequencing to analyze their transcriptomes. We found distinct differences in the populations. Principal component analysis (Fig. 5A) revealed that DCs broadly clustered based on infection state (PC1, 59.47% of variation) and subset (PC2, 29.66% of variation). We then assessed differential gene expression between infected and uninfected cDC1s and cDC2s. Pathway analysis using Ingenuity Pathway Analysis (IPA) was conducted. Many of the pathways significantly upregulated in infected cDC1s (Fig. 5B) and cDC2s (Fig. 5C) involved DC maturation and pathogen recognition, especially intracellular pathogens. Notably, cell death pathways were also upregulated upon infection. In addition, gene set enrichment analysis (GSEA) revealed that cDC1s (Fig. 5D) and cDC2s (Fig. 5E) from infected mice had enrichment of genes involved in apoptosis (Table S1) compared to uninfected counterparts, supporting our observations of *C. trachomatis*-induced apoptosis.

### *C. trachomatis* infection reduces the ability of dendritic cells to prime T cells

**Fig 6:**
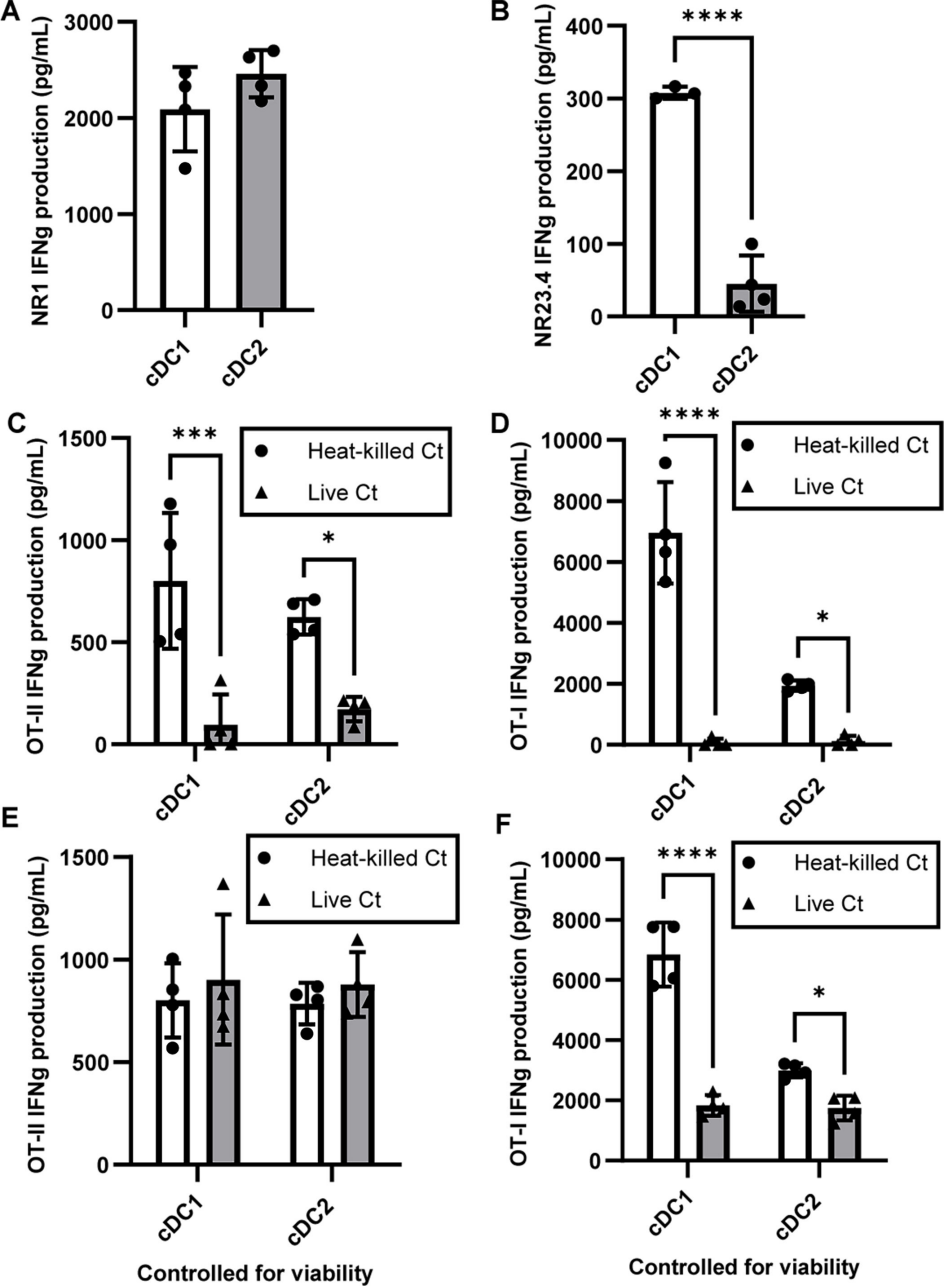
*C. trachomatis* infection reduces the ability of DCs to prime T cells. (A and B) Splenic DCs were sorted from C57BL/6 mice and incubated with *C. trachomatis*. DCs were then washed and co-cultured with *C. trachomatis*-specific (A) CD4+ or (B) CD8+ T cells for 3 days. Supernatant was collected, and IFNγ production was measured using ELISA. (C and D) Splenic DCs were isolated with CD11c microbeads and incubated with live or heat-killed *C. trachomatis* and ovalbumin (OVA). DCs were then sorted into subsets and co-cultured with (C) CD4+ OT-II or (D) CD8+ OT-I cells. (E and F) Splenic DCs were isolated using CD11c microbeads and incubated with live or heat-killed *C. trachomatis* and OVA. DCs were sorted into subsets by FACS, and the same number of live DCs was co-cultured with (E) OT-II or (F) OT-I cells. (A and B) *n* = 4, (C–F) *n* = 3, and data are representative of three independent experiments for all panels. panels A and B were analyzed with unpaired *t* test and panels C–F with two-way ANOVA and Sidak’s multiple comparisons test. *P < 0.05, ***P < 0.001, and ****P < 0.0001. Data are represented as mean ± SD.

Our next goal was to determine whether *C. trachomatis* affects the ability of DCs to prime T cells. We first investigated which of the two DC subsets are more efficient in generating antigen-specific CD8+ and CD4+ T cell responses upon *C. trachomatis* infection. We harvested *C. trachomatis*-specific CD4+ T cells and CD8+ T cells from TCR transgenic NR1 and NR23.4 mice, respectively, and co-cultured them with splenic cDC1s and cDC2s pulsed with *C. trachomatis*. To ensure that any differences in T cell activation observed were not due to *C. trachomatis* directly acting on T cells, DCs were washed to remove any *C. trachomatis* not taken up before T cells were added to the culture. Three days later, IFNγ production by T cells was measured by ELISA, while T cell activation and proliferation were measured by flow cytometry. We found that both DC subsets were equally efficient in stimulating CD4+ T cells (Fig. 6A; Fig. S2A and C), but cDC1s were significantly better at CD8+ T cell stimulation than cDC2s both in terms of activation and proliferation of T cells (Fig. 6B; Fig. S2B and C), suggesting that either cDC1 cross-presentation upon *C. trachomatis* uptake is required for antigen presentation to CD8+ T cells or *C. trachomatis* antigens can enter the cytosol of cDC1s but not cDC2s.

Given that live *C. trachomatis* reduces the viability of DCs, we then sought to determine whether it changes the ability of DCs to prime T cells. We incubated splenic cDC1s and cDC2s with ovalbumin (OVA) and heat-killed or live *C. trachomatis* before co-culturing the DCs with OVA-specific OT-II CD4+ T cells and OT-I CD8+ T cells. For both DC subsets, DCs that had been incubated with live *C. trachomatis* were significantly less able to stimulate OT-II and OT-I cells compared to controls incubated with heat-killed *C. trachomatis* (Fig. 6C and D; Fig. S2D and E). In line with what has been reported in the literature, both DC subsets were able to stimulate OT-II cells, while cDC1s were much more efficient at stimulating OT-I cells. To determine if reduction in DC viability was the only factor contributing to decreased T cell priming, we incubated cDC1s and cDC2s with OVA and heat-killed or live *C. trachomatis* and co-cultured the same number of live DCs from each sample with OT-II and OT-I cells. After controlling for the number of live DCs, DCs incubated with live *C. trachomatis* no longer showed a clear defect in OT-II priming compared to heat-killed *C. trachomatis* (Fig. 6E; Fig. S2F and G). However, DCs incubated with live *C. trachomatis* were still much less efficient in priming OT-I cells compared to heat-killed controls (Fig. 6F; Fig. S2F and G) even though the decrease in priming efficiency is less drastic than without controlling for DC viability. Aside from inducing DC death, other mechanisms may thus also play a role in the inhibition of CD8+ T cell priming by *C. trachomatis*. Nonetheless, taken together, our data suggest that reducing DC viability is part of the mechanism by which *C. trachomatis* infection decreases the ability of DCs to prime T cells, which may, in turn, be responsible for the inefficient T cell responses we see during chronic *C. trachomatis* infection.

### *C. trachomatis* infection induces death in human dendritic cells

**Fig 7:**
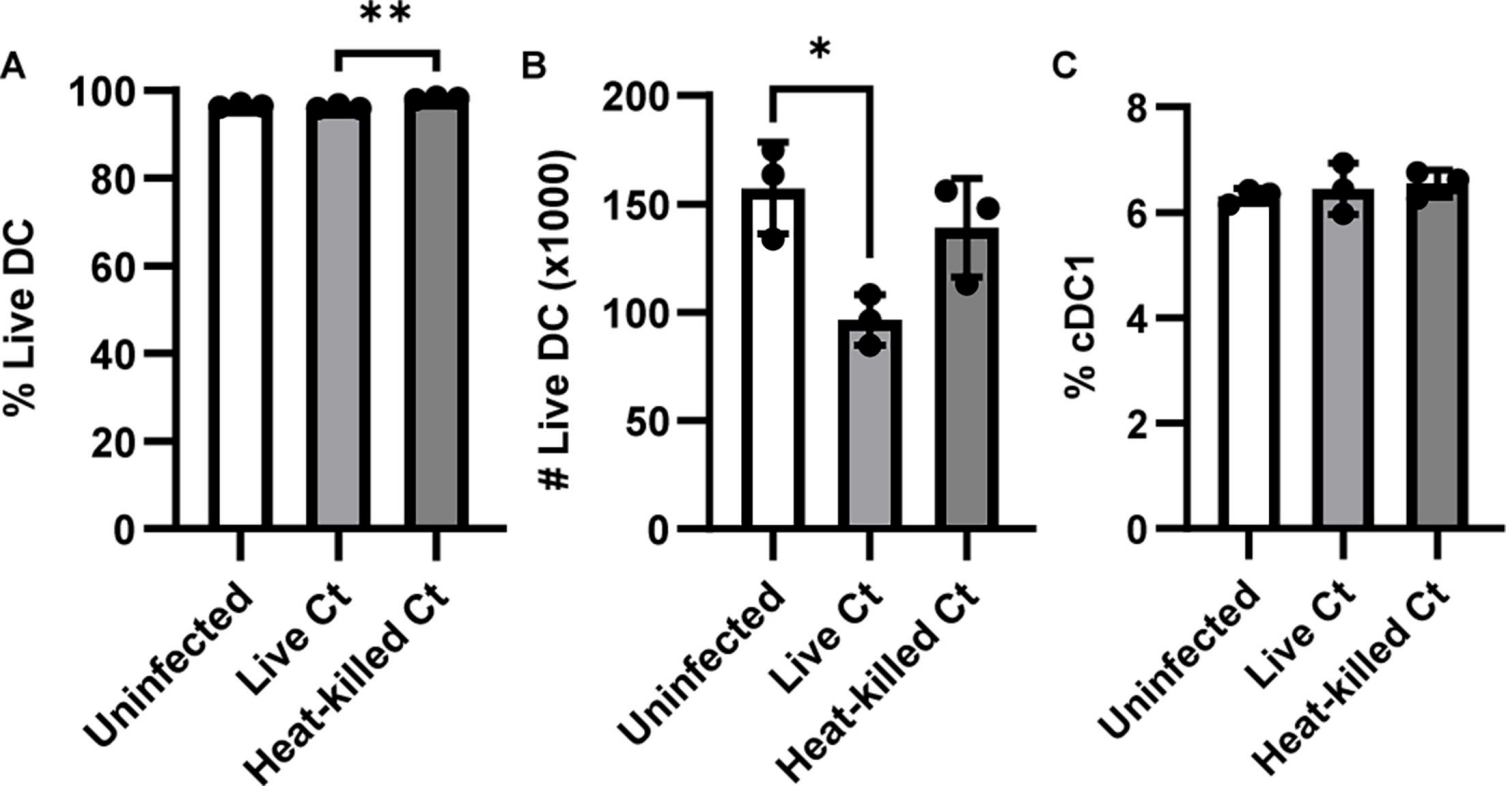
*C. trachomatis* infection induces death in human DCs. DCs were isolated from human buffy coats and infected with live or heat-killed *C. trachomatis* at an MOI of 5. The (**A**) percentage and (**B**) number of DCs (CD11c+ HLA-DR+) that remained viable 18 hours post-infection were measured with flow cytometry. (**C**) The percentage of live DCs that were cDC1s (Clec9A+ CD1c−). *P < 0.05 and **P < 0.01 analyzed with one-way ANOVA and Dunnett’s multiple comparisons test. Data are represented as mean ± SD.

Finally, to determine whether the *C. trachomatis*-induced death we observed in mouse DCs also happens in human DCs, we isolated DCs from human buffy coats. Isolated DCs were infected with heat-killed or live *C. trachomatis*, and cell viability was assessed via flow cytometry 18 hours post-infection (Fig. S3A). There were no major differences in the percentage of DCs that were alive between the groups (Fig. 7A; Fig. S3B), but samples infected with live *C. trachomatis* had a significantly lower number of live DCs compared to uninfected controls (Fig. 7B). It is possible that human DCs may die at a faster rate and were no longer detectable by flow cytometry at the point of analysis. As with our observations in mouse DCs, this decrease was attenuated with heat-killed *C. trachomatis*. However, unlike mouse DCs, there was no difference in the percentage of live DCs that were cDC1s between infected and uninfected samples (Fig. 7C). These data suggest that *C. trachomatis* infection can also induce DC death in human DCs, with both cDC1s and cDC2s equally affected.

## DISCUSSION

DCs are important antigen-presenting cells for stimulating a T cell response. Efficient T cell priming by DCs is required to clear pathogens and prevent chronic or repeated infections (12, 13). Previous work on *C. trachomatis* interactions with DCs has mostly used GMCSF-generated monocyte- or bone marrow-derived DCs (22–24, 26–28), which have characteristics distinct from *in vivo* DCs (30, 31) and may consist of a mix of macrophage- and DC-like cells. Hence, little is known about how cDC1s and cDC2s—the two DC subsets responsible for T cell priming *in vivo* (17)—respond to *C. trachomatis* infection and prime T cells. In this study, we first sought to investigate the effect *C. trachomatis* infection has on DCs. We showed that *C. trachomatis* induces cell death in both DC subsets *in vitro* and *in vivo*. Uptake of live *C. trachomatis* is required for DC death, and heat-killed *C. trachomatis* does not have the same effect. One explanation could be that certain secreted effectors produced by live *C. trachomatis* are causing DC death. It is possible that there are heat-labile factors in *C. trachomatis*—be it intracellular or on the cell surface—that are recognized by DCs to trigger cell death, but this is less likely since UV-inactivated *C. trachomatis* also showed attenuation in cell death induction (data not shown). Alternatively, *C. trachomatis* switches from the infectious elementary body stage to the more metabolically active reticulate body (RB) stage within a few hours of entering host cells (43), and it may be that factors inducing DC death are present in larger amounts during the RB stage. It is also possible that the process of intracellular infection started by live *C. trachomatis* causes more damage and stress to DCs, leading to cell death. Further work will have to be done to determine what factors produced by *C. trachomatis* are inducing DC death.

It is not unheard of for bacterial and viral infections to cause DC death. Pathogens may actively produce factors to induce cell death as a way of evading the immune system or DCs may undergo cell death to trigger inflammation and limit pathogen replication within the cell. Cell death may be in the form of apoptosis or more inflammatory modes of cell death such as pyroptosis. The intracellular bacteria *Legionella pneumophila*, for instance, stimulates caspase-1-dependent DC pyroptosis via the NOD-like receptor NAIP5 that recognizes bacteria flagellin, as well as caspase 3-dependent apoptosis by the activation of BAK and BAX (44). Both pyroptosis and caspase-3-mediated apoptosis are also known to occur during infection with *Francisella tularensis* when host AIM2 recognizes double-stranded DNA in the cytosol (37). Most previous studies of *Chlamydia* infection of human monocyte-derived or mouse BMDCs did not find any induction of DC death (24, 26, 28, 45–47), although cell death has been observed when the MOI is much higher (47), after prolonged infection of a few days (46), or when *Chlamydia* is introduced in the form of extrusions (26). A notable exception is Omosun et al. (27), who found that *Chlamydia* induces cell death in BMDCs in an NLRP3-inflammasome-dependent manner. Although it is unclear what exactly is causing the discrepancy, many papers used subtly different protocols for BMDC generation. GMCSF-generated BMDCs are a mix of macrophage- and DC-like cells (30, 31), and macrophages are known to at least somewhat allow productive *Chlamydia* replication (48, 49). Part of this discrepancy may, thus, be due to differences in the process of BMDC generation that resulted in different mixtures of cell types.

In our study, we used splenic DCs, which are more physiologically relevant and avoid the problem of heterogeneity posed by BMDCs. Like Omosun et al. (27), we found that *C. trachomatis* induces cell death in DCs. However, we cannot attribute this cell death to the NLRP3-inflammasome since caspase-1, the effector caspase downstream of NLRP3 signaling, is not required for DC death caused by *C. trachomatis*. Although knocking out caspases-1 and 11 had no impact on *C. trachomatis*-induced DC death, IL-1β, typically a hallmark of pyroptosis, is produced upon *C. trachomatis* infection in a CASP1/11-dependent manner. DCs may be driven to alternative pathways of cell death when pyroptosis is prevented. Alternatively, Zanoni et al. (50) found that DCs can produce IL-1β without dying under certain conditions. It is possible that the IL-1β production we observed is one such instance.

*Bak1/Bax* and *Casp3/7* knockout DCs, unlike *Casp1/11* knockouts, experience less cell death upon *C. trachomatis* infection. Rescue of cell death was not complete, which is unsurprising given the redundancies in cell death pathways. Nonetheless, together with data from Annexin V/PI staining and RNA sequencing, our results suggest that DC death is likely caspase-dependent apoptosis via the mitochondrial pathway. The activation of both CASP3/7 and CASP1/11 pathways is reminiscent of what has been observed during infection with other pathogens. We ruled out the involvement of AIM2—responsible for pyroptosis and apoptosis upon *Francisella tularensis* infection (37)—but there are many other possibilities left to be explored. Based on RNA sequencing of DCs from infected mice, we found that many pathways involved in anti-viral responses were also upregulated in addition to apoptosis and cell death pathways. This is consistent with the detection of infection by an intracellular pathogen. Considering that previous examination of *C. trachomatis* in monocyte-derived DCs and BMDCs has found productive replication in these cells, alongside a lack of cell death induction (24, 46, 47), the DC death we observed here could, therefore, be a double-edged sword for both the host and pathogen; there are less viable DCs to generate T cell responses against the bacteria, but at the same time, DCs cannot serve as productive host cells and do not help spread *C. trachomatis* systemically.

Given the induction of DC death by *C. trachomatis*, it stands to reason that infected DCs are also less able to stimulate T cells. Indeed, we found that DCs pulsed with OVA and live *C. trachomatis* are less able to stimulate OVA-specific CD4+ and CD8+ T cells. cDC1s were more affected than cDC2s by *C. trachomatis*-induced death and yet tend to be the more efficient subset in priming CD8+ T cells in other infection and tumor models where DCs are not productively infected by the pathogen and cross-presentation by cDC1s are thus required (18, 20, 21). If this is also true in *C. trachomatis* infection, then the increased cell death could partially explain why the CD8+ T cell response is less efficient than CD4+ T cell response. Steele et al. (23) found that BMDCs can cross-present both bacteria and infected cells to *C. trachomatis*-specific CD8+ T cells in a proteasome-dependent manner. However, no such study has been done on cDC1s and cDC2s, and it was previously unknown if *C. trachomatis* can infect cDC2s efficiently enough to generate cytosolic antigens that can be presented to CD8+ T cells. We found that either cross-presentation by cDC1s was indeed required for the stimulation of a CD8+ T cell response against *C. trachomatis* or *C. trachomatis* antigens can only enter the cytosol of cDC1s but not cDC2s. More work will have to be done to determine which possible explanation is true. Nevertheless, even if DCs are infected by the bacteria, the infection in cDC2s is not efficient enough to allow the presentation of cytosolic antigens to CD8+ T cells, preventing CD8+ T cell activation by cDC2s.

DCs were able to stimulate *C. trachomatis*-specific T cells despite *C. trachomatis*-induced cell death upon uptake. T cell stimulation may be carried out by the smaller number of DCs that manage to survive after taking up *C. trachomatis* or by DCs that have phagocytosed dead infected cells (23). This effect may be stronger *in vivo*, where DCs at the site of infection are not exposed to *C. trachomatis* all at once, and may explain why the CD4+ T cell response in mice is enough to clear *C. trachomatis* infection even though a significant defect in CD4+ T cell priming is observed *in vitro*. The strict requirement of cDC1s for CD8+ T cell priming means that the higher death rate of cDC1s, in addition to the fact that cDC1s are fewer in numbers than cDC2s *in vivo*, could contribute to the defective CD8+ T cell response in mice compared to CD4+ T cells. However, *in vivo* studies will need to be carried out to ascertain if this is the case.

We also wanted to know if preventing DC death would rescue the defect in T cell priming. Unfortunately, the DC-T cell co-culture experiments we conducted with *Bak1/Bax* and *Casp3/7* knockout DCs were inconclusive (data not shown), probably due to a combination of technical difficulties in getting enough DCs and the fact that there was only a partial prevention of cell death in knockouts, leading to smaller effect sizes. As an alternative, we co-cultured the same number of viable heat-killed or live *C. trachomatis*-infected DCs with T cells to control for the difference in DC viability. Interestingly, controlling for viability rescued the defect in CD4+ T cell but not CD8+ T cell priming, even though the defect in CD8+ T cell priming was less drastic than without controlling for the numbers of live DCs. This suggests that other factors aside from cell death are also playing a role in preventing CD8+ T cell activation. Indeed, Fankhauser et al. (29) showed that *C. trachomatis* can restrict CD8+ T cell response by upregulating PD-L1 on DCs and epithelial cells. We found from our RNA sequencing data that PD-L1 is but one of many co-inhibitory genes upregulated (data not shown). Further work is required to validate these genes and investigate their role in inhibiting T cell priming, as well as what makes CD8+ T cells more susceptible to them. The final effect on T cell priming most likely results from an interplay of DC viability, co-stimulatory and co-inhibitory molecules, receptors expressed on T cells, as well as other chemokines and cytokines expressed by DCs.

Consistent with our experiments using mouse DCs, cell death is also induced by *C. trachomatis* in human DCs. Unlike mouse DCs, cDC1s and cDC2s were equally affected by *C. trachomatis*-induced cell death. It is worth noting that also unlike mouse DCs, where cDC1s are much better at cross-presentation to CD8+ T cells than cDC2s, the two DC subsets in humans have similar cross-presentation capacities (51). Mice are able to generate an effective CD4+ T cell response against *C. trachomatis* infection (6, 7), while most humans develop only a partial immunity at best (5). More work on the interactions between *C. trachomatis*-infected human DCs and T cells will have to be done to find out why this is the case and if it is related to the differential response of mouse and human DC subsets to *C. trachomatis*.

In summary, our study shows that *C. trachomatis* induces DC death and reduces the ability of DCs to prime T cells. CD8+ T cell priming is impaired to a greater extent than CD4+ T cells, which may be part of the reason why mice have a less efficient CD8+ T cell response against *C. trachomatis*. Our work provides insight into how T cells are activated during natural infection, which is ultimately important for designing a vaccine that can generate protective T cells for an effective immune response upon infection.

## MATERIALS AND METHODS

### Growth and isolation of bacteria

*Chlamydia trachomatis* serovar L2 (434/Bu; ATCC) and *C. trachomatis* GFP (52) (generously provided by Isabelle Derré and Raphael Valdivia) were propagated in McCoy cell monolayers as described previously (53, 54). Aliquots of purified elementary bodies were stored at –80°C in 250 mM sucrose-phosphate-glutamate (SPG) buffer and thawed immediately prior to use. Where indicated, heat-killing was achieved by incubating *C. trachomatis* for 20 minutes at 65°C.

### Mice

C57BL/6J, B6.SJL-Ptprc^a^ Pepc^b^/BoyJ (CD45.1), B6J.129(Cg)-^Igs2tm1.1(CAG-cas9*)Mmw^/J, B6.129P2-Aim2^Gt(CSG445)Byg^/J, and B6N.129S2-Casp1^tm1Flv^/J female mice were purchased from The Jackson Laboratory (Bar Harbor, ME, USA). NR1 (55) and NR23.4 (56) transgenic mice expressing TCRs specific for *C. trachomatis* antigens Cta1_133-152_ and CrpA_63-71_, respectively, have been previously described. TCR transgenic OT-I mice that recognize OVA_257-264_ and OT-II mice that recognize OVA_323-339_ were kindly provided by Ulrich von Andrian. All mice were housed in the Harvard Medical School Center for Animal Resources and Comparative Medicine.

### Dendritic cell isolation and infection

Spleens were harvested from mice, and single-cell suspensions were created using gentleMACS Dissociator (Miltenyi Biotec) as per the manufacturer’s instructions (program m_spleen_01). Addition of collagenase digestion did not significantly enhance the yield of viable DCs and was thus not utilized to reduce the time the DCs spent between harvesting from mice and subsequent experiments. Red blood cells were lysed with tris-ammonium-chloride, and splenocytes were infected with *C. trachomatis* and cultured in RPMI (Invitrogen) supplemented with 10% FBS, L-glutamine, HEPES, 50 mM 2-mercaptoethanol, and 20 ng/mL murine GMCSF (Peprotech). Where indicated, dendritic cells were isolated from splenocytes using CD11c MicroBeads UltraPure (Miltenyi Biotec) following the manufacturer’s protocol. Unless otherwise stated, splenocytes were infected with *C. trachomatis* at an MOI of 2, while isolated DCs were infected at an MOI of 5.

### Heparin incubation

Inhibition of *C. trachomatis* uptake by dendritic cells was achieved by pre-incubating *C. trachomatis* in PBS containing 100 U/mL of heparin sodium salt (Millipore Sigma) for 1 hour at 4°C before adding bacteria to cells (33, 34).

### Dendritic cell burden analysis

Isolated CD11c+ splenic dendritic cells were infected with *C. trachomatis* at an MOI of 50 (24, 56). Two hours post-infection, cells were FACS sorted on FACS Aria (BD) into cDC1 (lineage− CD11c+ MHC I-A^b^+ CD8+) and cDC2 (lineage− CD11c+ MHC I-A^b^+ CD11b+) subsets. Bacterial burden was determined through quantitative PCR as previously described (57). Briefly, DNA was extracted from samples using Quick-DNA Microprep kit (Zymo Research). *Chlamydia* 16S DNA and mouse GAPDH were quantified using primer pairs and dually labeled probes (IDT, San Jose, CA, USA, and Applied Biosciences).

### T cell isolation and co-culture

CD4+ T cells were isolated from secondary lymphoid organs of NR1 and OT-II mice, and CD8+ T cells from NR23.4 and OT-I mice. Cells were purified using Dynabeads Untouched Mouse CD4 Cells kit or CD8 Cells kit (Invitrogen). For NR1 and NR23.4 cells, sorted dendritic cells (5,000 cells per well in 96-well plate) were incubated with *C. trachomatis* at an MOI of 50. Three hours post-infection, dendritic cells were washed twice and co-incubated with the isolated T cells for 3 days at a ratio of 1:5. For OT-I and OT-II cells, CD11c+ DCs were incubated with live or heat-killed *C. trachomatis* at an MOI of 50 and/or ovalbumin (50 µg/mL), sorted into DC subsets, and co-cultured at a ratio of 1:10. Supernatant was then collected, and IFNγ production was analyzed with ELISA. To measure T cell proliferation and activation, T cells were pre-stained with eBioscience Cell Proliferation Dye eFluor 450 (Invitrogen) following the manufacturer’s protocol before incubation with DCs and analyzed with flow cytometry 3 days post-infection.

### Infection of mice and preparation of tissue

Mice were injected intravenously via the tail vein with 10^7^ inclusion-forming units of *C. trachomatis* in 200 µL SPG buffer or SPG buffer alone (8). At specific times post-infection, spleens were harvested, and single-cell suspensions were prepared by grinding the tissue between frosted microscope slides.

### Flow cytometry

All antibodies were purchased from BioLegend unless otherwise noted. After isolation, cells were pre-incubated with anti-mouse CD16/CD32 (2.4G2) and stained with fluorochrome-conjugated antibodies against mouse CD11c (clone N418), MHC class I-A^b^ (clone AF6-120.1), CD11b (clone M1/70), CD8a (clone 53-6.7), F4/80 (clone BM8), CD4 (clone GK1.5), CD3 (clone 17A2), CD45.1 (clone A20), CD45.2 (clone 104), TCRb (clone H57-597), CD49b (clone HMa2), IgM (clone RMM-1), CD2 (clone RM2-5), CD117 (clone S18020A), TER-119 (clone TER-119), Gr-1 (clone RBC-8C5), CD3e (clone 145-2C11), CD5 (clone 53–7.3), B220 (clone RA3-6B2), and a Live/Dead Fixable Aqua Dead Cell Stain Kit (Invitrogen) or Zombie NIR Fixable Viability Kit (BioLegend). For the detection of apoptotic cells, cells were washed twice after antibody staining and incubated with Annexin V (BioLegend) and washed, followed by propidium iodide (BioLegend) just before flow analysis. For human DCs, cells were pre-incubated with Human BD Fc block (BD Pharmingen), followed by staining with anti-human CD11c (clone 3.9), HLA-DR (clone L243), CD1c (clone L161), Clec9A (clone 8F9), and Zombie NIR fixable viability kit. The absolute cell number was determined using AccuCheck counting beads (Invitrogen). Data were collected on a LSR II or Symphony A1/A5 (BD Biosciences) and analyzed using FlowJo (Tree Star, Ashland, OR, USA).

### RNA sequencing and analysis

Cells were sorted and processed for RNA sequencing according to the Immgen protocol for ultra-low-input RNA-seq analysis as previously described (58). Briefly, spleens from mice were harvested and processed into single-cell suspensions. RNA sequencing was conducted on biological triplicates of 1,000 cDC1s and 1,000 cDC2s that were double sorted by MoFlo Astrios. Smart-Seq2 library preparation, sequencing (59, 60), and transcript quantification (61) were then performed as previously described. Reads were analyzed using Ingenuity Pathway Analysis (Qiagen) and RStudio (PBC, MA, USA). Gene set enrichment analysis (62, 63) was performed using default settings with software version 4.3.2 and canonical pathways gene sets from WikiPathways database. The sequencing data are available in the BioProject database under accession number PRJNA1136144.

### ELISA

For the detection of IL-1β secretion by dendritic cells, IL-1β ELISA was conducted using an IL-1 beta Mouse Uncoated ELISA Kit (Invitrogen) following the manufacturer’s instructions. Standard IFNγ ELISA protocols were used to measure T cell activation following co-culture. Briefly, plates were coated with 1 µg/mL of anti-mouse IFNγ (clone R46A2, BD) in 0.1 M bicarbonate buffer. Plates were then blocked with PBS containing 1% BSA and washed in PBS with 0.05% Tween. After incubation with samples, plates were incubated with 0.5 µg/mL biotinylated anti-mouse IFNγ (clone XMG1.2, BioLegend) followed by Streptavidin-HRP conjugate (BD Pharmingen) diluted 1:2,500. HRP activity was detected by adding tetramethylbenzidine peroxidase substrate, and quantification was performed by spectrophotometric measurement of absorbance at 450 and 570 nm after reactions were stopped by 1 M sulfuric acid.

### Guide RNA design and cloning

Plasmids for CHIME were generated as previously described (41, 42). Briefly, sgRNA nucleotides were designed using the Broad CRISPR algorithm. *Bak1*: 5′-GCCCACAGCCTATTTAAGAG-3′; *Bax*: 5′-GCTGATGGCAACTTCAACTG-3′; *Casp1*: 5′-TCAACTTGAGCTCCAACCCT-3′; *Casp3*: 5′-CATGCAGAAAGACCATACAT-3′; *Casp11*: 5′-TGAAGACTTAGGCTACGATG-3′; *Casp7*: 5′-GGACGGTTACTTCAAAACCC-3′; and *Cd205*: 5′-GTCACGAAACTCCATAATGG-3′. To target two genes simultaneously, sgRNA nucleotides were cloned into the C-CHIME plasmid vector pXPR_219 (Addgene ID# 164559, kindly provided by Arlene Sharpe) using BsmBI (New England Biosciences) followed by BfuAI (New England Biosciences) restriction digestion.

### Generation of lentivirus

Lentiviral constructs were generated by transfecting 293T cells (ATCC) grown in DMEM (Corning) supplemented with 10% FBS with pMD2.G (Addgene #12259), psPAX2 (Addgene #12269), and the edited pXPR_219 plasmid. Seventy-two hours post-transfection, lentiviruses in media were harvested and concentrated using ultracentrifugation.

### Creation of bone marrow chimeric mice

Bone marrow chimeras were generated as previously described (41). Briefly, tibias, femurs, hips, and spines were harvested from Cas9-expressing CD45.2^+/+^ mice, and LSK (lineage−Sca-1^+^Kit^+^) cells were isolated by CD117 Microbeads kit (Miltenyi Biotec) followed by FACS sorting. LSK cells were spin transduced with lentiviral constructs on a Retronectin-coated plate (TakaraBio) and transferred intravenously into lethally irradiated Cas9-expressing CD45.2^+/−^ recipients.

### Human dendritic cell isolation

Fresh buffy coats of healthy blood donors were purchased from Boston Children’s Hospital Blood Donor Center (Boston, MA,USA). Mononuclear cells were isolated using Lympholyte-H Cell Separation Media (Cedarlane), and dendritic cells were purified using an EasySep Human Myeloid DC Enrichment Kit (StemCell Technologies) following the manufacturer’s instructions. Cells were then infected with *C. trachomatis* and cultured in RPMI (Invitrogen) supplemented with 10% FBS, L-glutamine, HEPES, 50 mM 2-mercaptoethanol, and 500 IU/mL human GMCSF (Peprotech).

### Statistical analysis

Statistical analysis was performed using Prism (GraphPad) unless otherwise stated. Differences were considered statistically significant if the *P* value was less than 0.05. *P < 0.05; **P < 0.01; ***P < 0.001; and ****P < 0.0001. Data are represented as mean ± SD.

## SUPPLEMENTAL MATERIAL

Supplemental material iai.00402-24-s0001.pdf Fig. S1 to S3; Table S1.

ASM does not own the copyrights to Supplemental Material that may be linked to, or accessed through, an article. The authors have granted ASM a non-exclusive, world-wide license to publish the Supplemental Material files. Please contact the corresponding author directly for reuse.
