# Supplementary material for: Chlamydia trachomatis impairs T cell priming by inducing dendritic cell death
Source: Infect Immun. 2025 Jan 8;93(2):e00402-24. doi: 10.1128/iai.00402-24 (PMC11834465; doi:10.1128/iai.00402-24)
Supplement: Supplemental material — Fig. S1 to S3; Table S1. [file iai.00402-24-s0001.pdf]

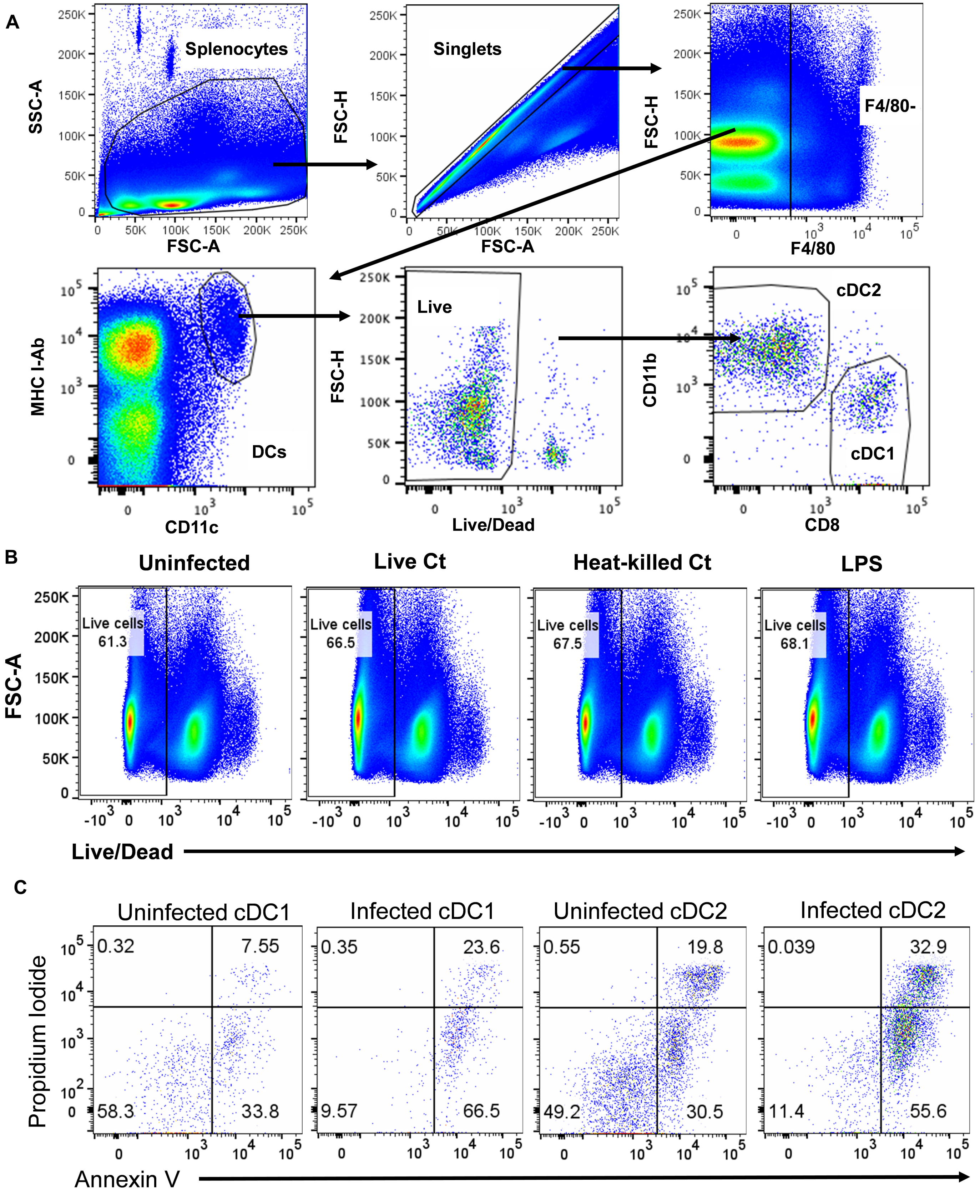

Supplemental Figure 1: *C. trachomatis*-infected DCs experience increased apoptosis. (A) Gating strategy. Splenocytes were isolated from C57BL/6 mice and infected with live or heat-killed *C. trachomatis* at a MOI of 2, or LPS control (100ng/mL). Eighteen hours post-infection, cDC1s (gated on F4/80- CD11c+ MHC I-Ab+ CD8+ CD11b- cells) and cDC2s (F4/80- CD11c+ MHC I-Ab+ CD8- CD11b+) were analyzed by flow cytometry. (B) The viability of non-DCs in the samples. (C) Splenic cells were isolated from C57BL/6 mice and co-cultured with *C. trachomatis*. After eighteen hours, samples were stained with FITC-Annexin V and propidium iodide (PI).

Supplemental Table 1. List of genes in apoptosis pathway upregulated in infected cDC1s compared to uninfected cDC1s and in infected cDC2s compared to uninfected cDC2s as analyzed by GSEA.

Genes enriched in infected cDC1s		Genes enriched in infected cDC2s	
**SYMBOL**	**RANK METRIC SCORE[1]**	**SYMBOL**	**RANK METRIC SCORE[1]**
Irf7	4.391	*Irf7*	3.727
Tnfsf10	2.218	*Irf1*	2.024
Irf4	1.811	*Casp3*	1.999
Traf1	1.577	*Tnfrsf1b*	1.748
Apaf1	1.560	*Nfkbib*	1.454
Nfkbib	1.509	*Fas*	1.442
Irf1	1.315	*Casp4*	1.441
Mdm2	1.315	*Cflar*	1.432
Casp3	1.301	*Tnfsf10*	1.422
Traf2	1.283	*Bcl2l11*	1.329
Birc2	1.279	*Birc2*	1.288
Cflar	1.249	*Nfkbia*	1.276
Casp4	1.239	*Apaf1*	1.241
Ikbkb	1.227	*Mdm2*	1.128
Tnfrsf1b	1.227	*Ikbkb*	1.098
Fas	1.179	*Lta*	1.058
Ripk1	1.135	*Traf2*	1.035
Tnfrsf25	1.124	*Bcl2l1*	1.019
Rela	1.121	*Traf1*	1.017
Traf3	1.055	*Mcl1*	0.888
Lta	1.049	*Casp9*	0.844
Mcl1	1.021	*Casp8*	0.834
Pik3r1	1.015	*Casp1*	0.830
Irf6	1.008	*Birc3*	0.726
Nfkbie	1.000	*Tnfrsf25*	0.712
Casp7	0.937	*Tnfrsf10b*	0.633
Bcl2l1	0.930	*Irf5*	0.633
Nfkbia	0.864	*Prf1*	0.631
Ikbkg	0.848	*Irf6*	0.624
Birc3	0.810	*Irf2*	0.609
		Ripk1	0.608

[1] A positive rank metric score indicates a gene's positive correlation to the pro-apoptotic phenotype observed in infected DCs.

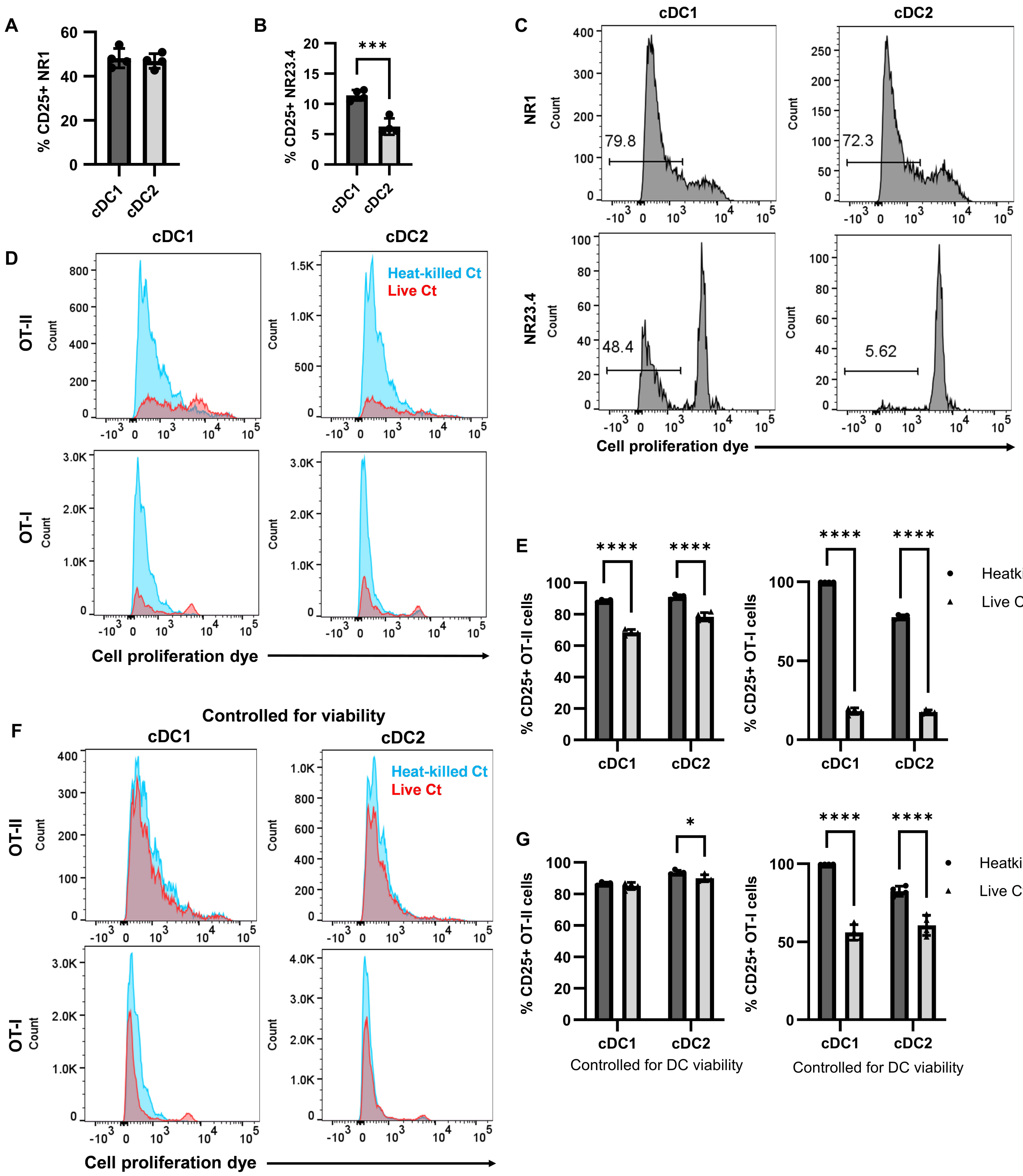

Supplemental Figure 2: *C. trachomatis* infection reduces the ability of DCs to prime T cells. (A-C) Splenic DCs were sorted from C57BL/6 mice and incubated with *C. trachomatis*. DCs were then washed and co-cultured with *C. trachomatis*-specific (A) CD4+ or (B) CD8+ T cells pre-stained with cell proliferation dye. (D-E) Splenic DCs were isolated with CD11c microbeads and incubated with live or heat-killed *C. trachomatis* and OVA. DCs were sorted into subsets and co-cultured with CD4+ OT-II or CD8+ OT-I cells. (F-G) Splenic DCs were isolated and incubated with *C. trachomatis* and OVA, then sorted into subsets and the same number of live DCs were co-cultured with OT-II or OT-I cells. T cell activation (A-B, E, G) and proliferation (C, D, F) were measured using flow cytometry. Data representative of 3 independent experiments. (A-B) were analyzed with unpaired t test and (E, G) with two-way ANOVA and Sidak's multiple comparisons test. *$P < 0.05$, *** $P < 0.001$, ****$P < 0.0001$. Data are represented as mean ± SD.

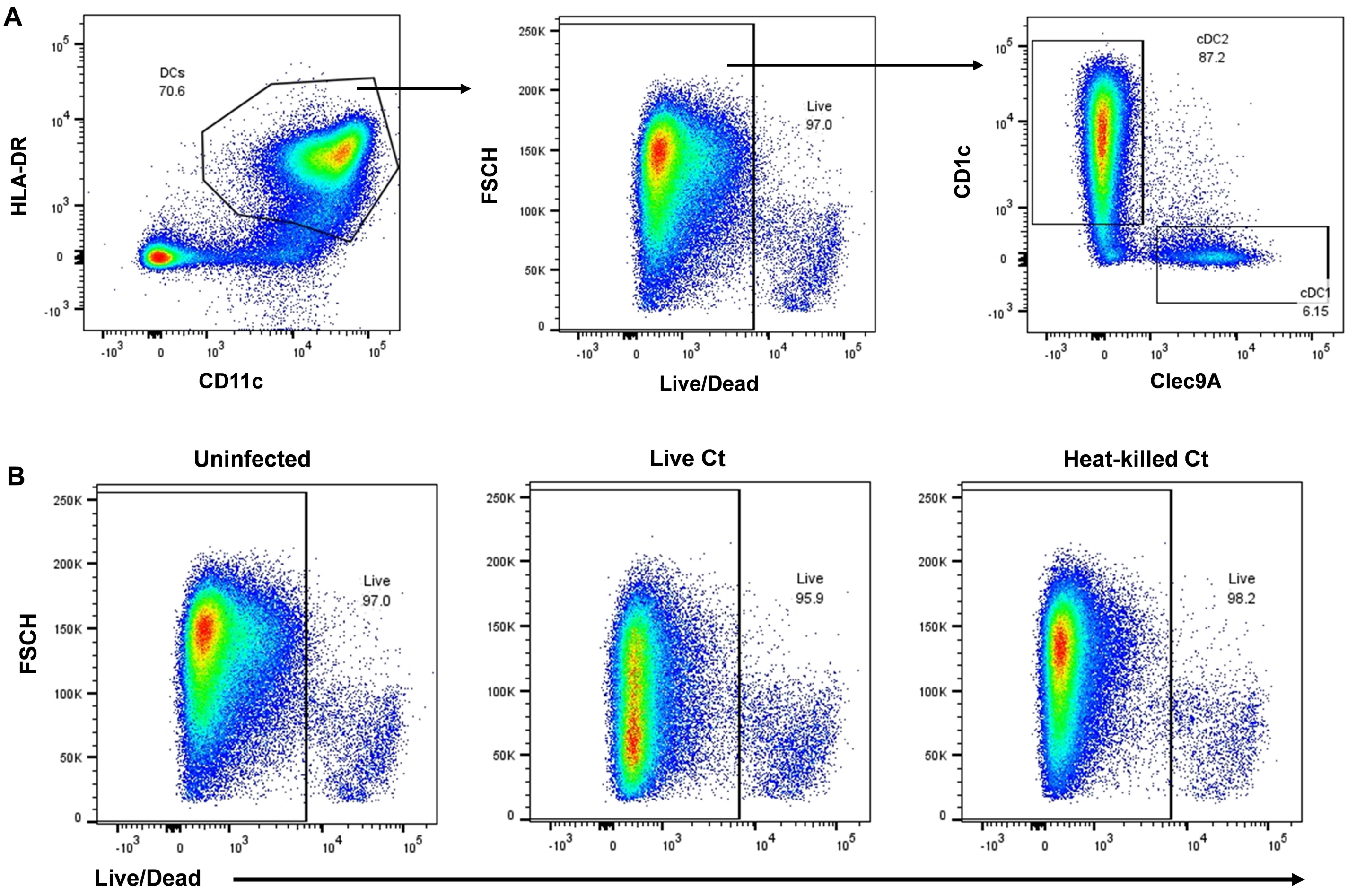

Supplemental Figure 3: ***C. trachomatis*** **infection induces death in human DCs.** (A-B) DCs were isolated from human buffy coats and infected with live or heat-killed *C. trachomatis* at a MOI of 5. DCs (CD11c+ HLA-DR+) that remained viable eighteen hours post-infection were analyzed with flow cytometry. (A) Gating strategy. (B) Percentage of DCs that were viable.
